# Generally Applicable Transformation Protocols for Fluorescent Nanodiamond Internalization into Cells

**DOI:** 10.1038/s41598-017-06180-5

**Published:** 2017-07-19

**Authors:** Simon R. Hemelaar, Kiran J. van der Laan, Sophie R. Hinterding, Manon V. Koot, Else Ellermann, Felipe P. Perona-Martinez, David Roig, Severin Hommelet, Daniele Novarina, Hiroki Takahashi, Michael Chang, Romana Schirhagl

**Affiliations:** 1 Department of Biomedical Engineering, University of Groningen, University Medical Center Groningen, Antonius Deusinglaan 1, 9713 AW Groningen, Netherlands; 2 European Research Institute for the Biology of Ageing, University of Groningen, University Medical Center Groningen, Antonius Deusinglaan 1, 9713 AW Groningen, Netherlands; 3 Department of Physics, ETH-Zurich, Otto Stern Weg 1, Zurich, Switzerland

## Abstract

Fluorescent nanodiamonds (FNDs) are promising nanoprobes, owing to their stable and magnetosensitive fluorescence. Therefore they can probe properties as magnetic resonances, pressure, temperature or strain. The unprecedented sensitivity of diamond defects can detect the faint magnetic resonance of a single electron or even a few nuclear spins. However, these sensitivities are only achieved if the diamond probe is close to the molecules that need to be detected. In order to utilize its full potential for biological applications, the diamond particle has to enter the cell. Some model systems, like HeLa cells, readily ingest particles. However, most cells do not show this behavior. In this article we show for the first time generally applicable methods, which are able to transport fluorescent nanodiamonds into cells with a thick cell wall. Yeast cells, in particular *Saccharomyces cerevisiae*, are a favored model organism to study intracellular processes including aging on a cellular level. In order to introduce FNDs in these cells, we evaluated electrical transformation and conditions of chemical permeabilization for uptake efficiency and viability. 5% DMSO (dimethyl sulfoxide) in combination with optimized chemical transformation mix leads to high uptake efficiency in combination with low impact on cell biology. We have evaluated all steps in the procedure.

## Introduction

In recent years fluorescent nanodiamonds (FNDs) have gained a great deal of attention^1^. Their stable fluorescence permits long-term tracking and their magneto-optical behavior allows them to be used as sensors for different properties in the environment, owing to Nitrogen Vacancy (NV) centers inside the nanodiamond particle^2^. Diamond defects are so sensitive that nanoscale temperature measurements (accuracies down to 1 mK)^3^ or measuring the magnetic fields of single electron spins^4^ are possible. Other advantages are the biocompatibility of FNDs and their excellent inertness while the surface is modifiable^5–7^. However, to fully deploy their potential for bioapplications the diamond particles need to enter cells. Some mammalian cells, for instance HeLa cells or macrophages, readily ingest diamond nanoparticles without surface modification or chemical stimulus^8–11^. A few selected other cells show a similar behavior and readily take up particles^12–14^.

In case the cells of interest do not readily ingest particles, only a limited set of methods is currently available. One way is to inject cells with nanodiamonds using a silicon nanowire^15^. This was achieved for human embryonic fibroblast WS1 cells. Tzeng *et al*. introduced BSA-coated diamonds by electroporation into (HeLa) cells^16^. An additional way to control diamond uptake is to treat the cells with different chemicals, such as NaN3, sucrose or filipin^17, 18^.

Most of these techniques have only been applied to HeLa cells or similar and none of these methods have been tested to achieve uptake in non-mammalian cells. For multicellular organisms or very large cells (e.g. egg cells), nanodiamonds can simply be injected^19^. However, this approach is quite invasive and not applicable for average-sized single cells.

In this study we focused on *Saccharomyces cerevisiae*, which is considered one of the most important model organisms to study a wide range of biological processes. In particular, they are a favored model organism to study ageing. Their high turnover rate and the suitability for genetic manipulation^20^, as well as the many basic biological processes which are highly conserved from yeast to humans, demonstrate their relevance. Additionally, the fact that young and old cells can be separated relatively easy also offers great potential to study ageing. Furthermore, yeast is widely used in food industries as well as in biotechnology to produce different pharmaceutical products^21–23^. Unlike human cells, yeast cells have a thick cell wall. In order to introduce FNDs into these cells, this obstacle needs to be overcome.

In this study, we show for the first time broadly applicable approaches, which enable the nanodiamond particles to enter cells which do not readily ingest particles. We demonstrate different uptake methods, which were optimized with regard to their success rate as well as their impact on cell viability.

## Results

### FND uptake

**Figure 1:**
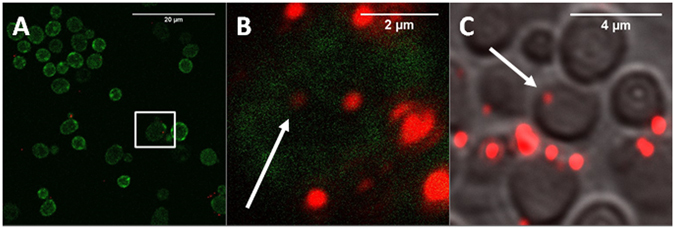
Qualitative analysis of uptake of FNDs by Hxt6-GFP-expressing yeast cells. (**A**) Overview of FNDs and Hxt6-GFP-expressing cells after treatment with TMIX, incubation at 42 °C and treatment with 5% DMSO. (**B**) Close up of the boxed cell from A, with diamond particles inside (for better visibility of the diamond particles the contrast of the red channel has been increased). The arrow shows a particle that was not at any place associated with the cell membrane. (**C**) Embedded and sectioned (approximately 0.8 µm thickness) cells, visualized using Differential Interference Contrast. The diamond particle indicated by the arrow is internalized.

**Figure 2:**
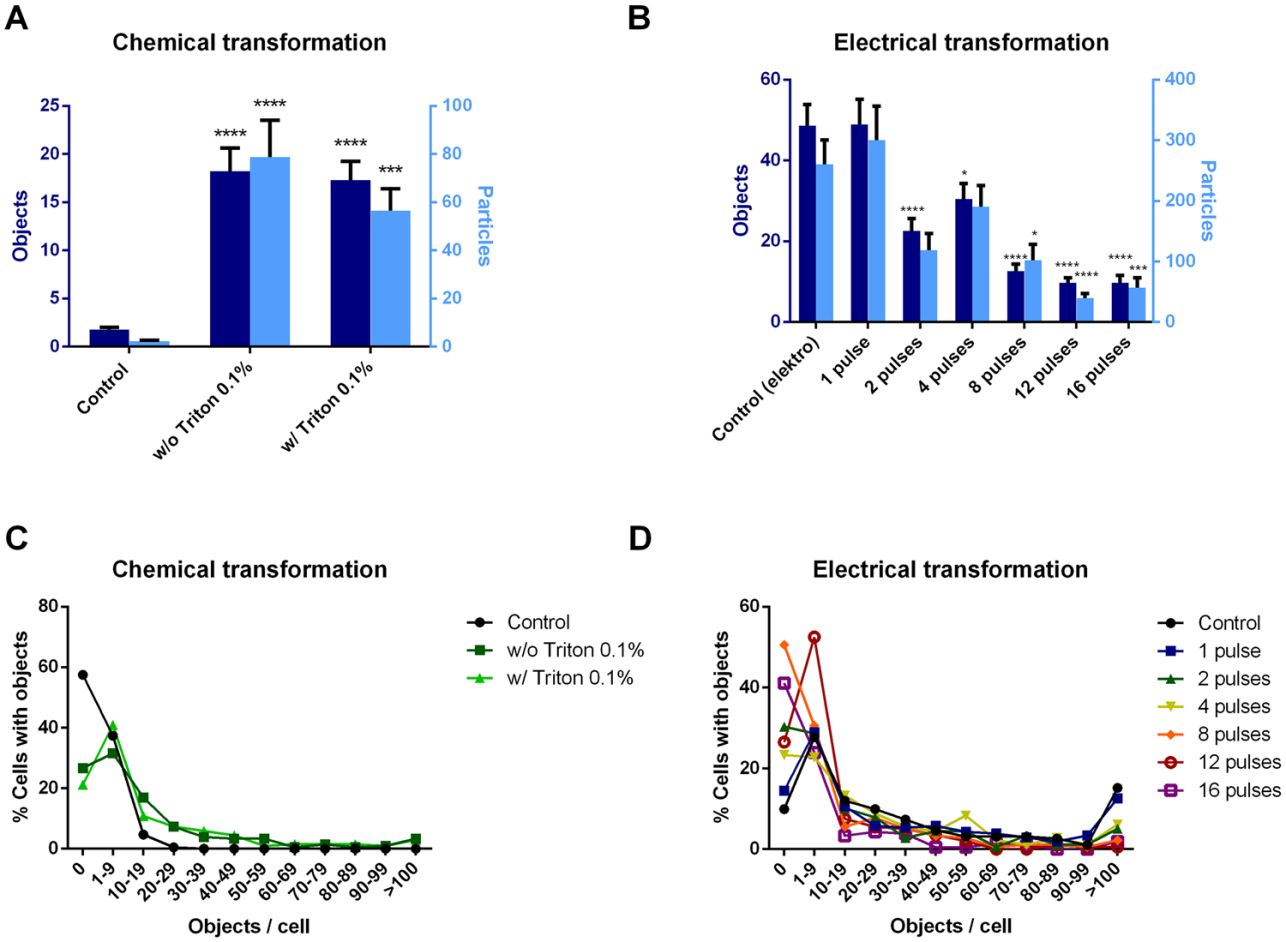
Quantitative analysis of FND uptake by Hxt6-GFP-expressing *Saccharomyces cerevisiae* cells. The amount of objects (adjacent FND positive pixels are counted as an object) and particles (an object can also be an aggregate consisting of more than one particle) is estimated through our home written FIJI protocol (see Method section: FND uptake quantification). For all situations, 2 times approximately 100 cells were selected and cells with obvious large aggregates on the exterior were excluded post-hoc. (**A** and **B**) show the absolute numbers for both types of transformations. In (**C** and **D**) a grouped distribution of the percentage of cells carrying a range of objects is shown. Significance is tested compared to the control situation. *p < 0.05, ***p < 0.001, ****p < 0.0001.

The uptake of FNDs in yeast cells was confirmed both by confocal microscopy and additionally by sectioning of embedded samples (Fig. 1). The absolute amount of FND uptake could not be measured, as a diffraction limited spot is at least one particle but could also be a small cluster. An estimation to value the amount of particles was modelled through a home written FIJI based script (see methodology section: FND uptake quantification). Fig. 2 shows the uptake of objects (adjacent FND positive pixels) and particles. The amount of particles is estimated by the total intensity of the aforementioned objects. In Fig. 2A,B the absolute amount of objects and particles are shown as estimated by our FIJI protocol. The control should be considered as a background measurement, as it is not possible to completely eliminate noise and the signal of membrane bound diamond particles due to limited Z-resolution. The chemical transformation is highly successful in introducing diamond into yeast cells. Notably, the electroporation protocol actually reduced uptake of diamond particles after multiple pulses, possibly due to the reduced viability of the cell. Dead cells lose their membrane integrity and internalized diamond particles are washed away by the washing steps in the electroporation protocol. In Fig. 2C,D we show a distribution of the percentage of cells with a certain number of internalized objects. For both transformation methods, there is a larger percentage of cells with a high amount of particles compared to the control.

### Biocompatibility

**Figure 3:**
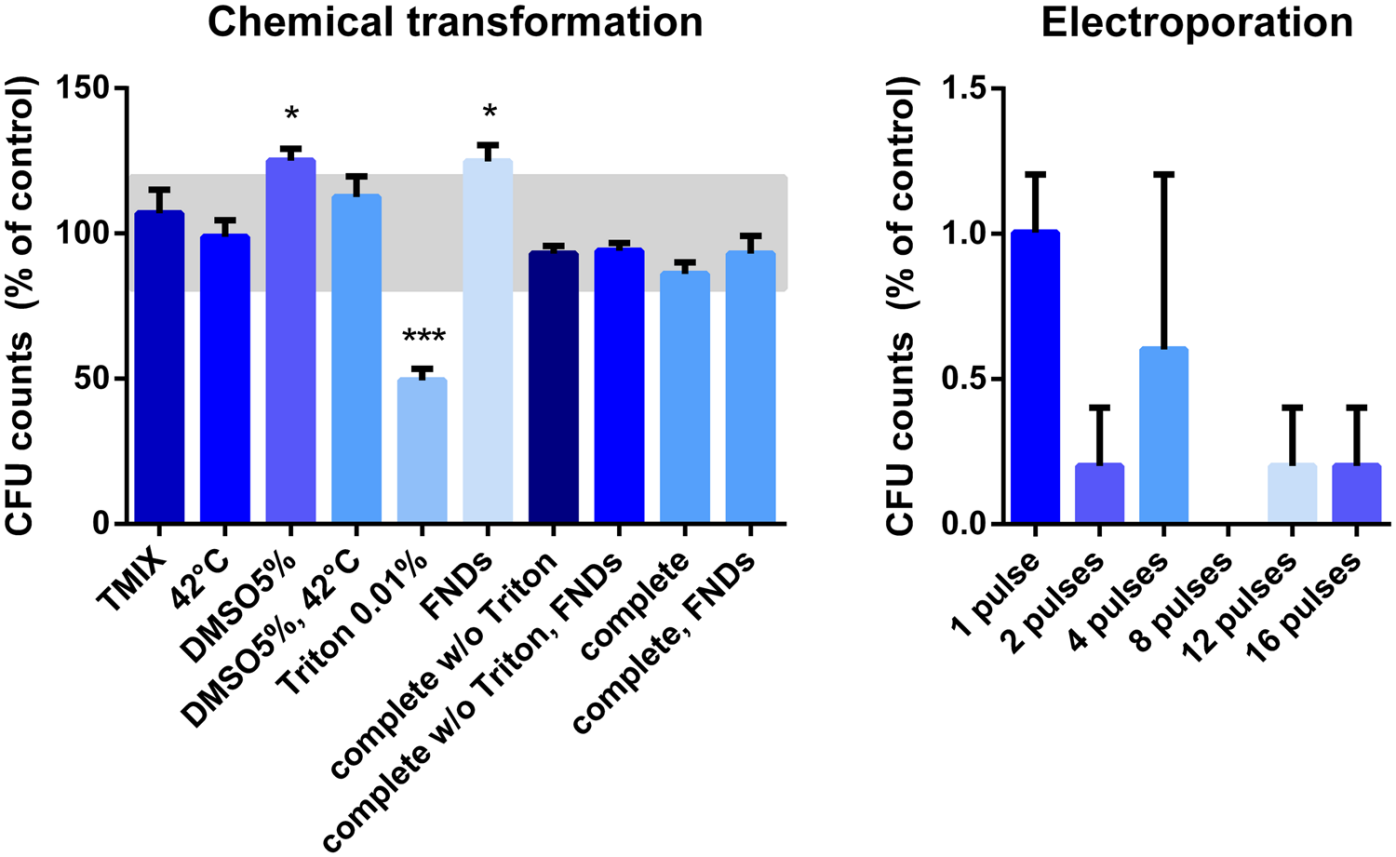
Survival of yeast cells after performing the interventions and different steps in the transformation protocols separately. Colony forming units (CFUs) are a measure for the survival of viable cells after either the chemical transformation or the electroporation protocol. (**A**) The greatest reduction of viability occurs after the addition of 0.01% Triton. The addition of FNDs to the treatment does not decrease the viability. Bars represent averages of triplicates out of two independent experiments. An area of +/−20% around 100% is deemed as ‘normal viability,’ (**B**). Electroporation reduces viability by a factor 10^2^. In all samples FNDs were also added. In the case of 8 pulses, cell viability was completely reduced. For the electroporation protocol all decreases were significant (p < 0.0001). Bars represent averages of replicates out of two independent experiments, error bars show the Standard Error of the Mean. Significance is tested compared to the control situation. *p < 0.05, ***p < 0.001.

The impact of the different interventions involved in the transformation methods on cell viability were analyzed by counting the amount of colony forming units (CFUs) after the interventions. The CFUs represent the amount of viable cells that survived the transformation. Since the cells not only need to be alive but also be able to proliferate, CFUs are generally considered to be the most stringent form of viability measurements^24^. For the chemical transformation technique, the different steps were tested separately and most of them were shown to have a low impact on the viability (Fig. 3A). Only the use of 0.01% Triton negatively affected the viability of the cells. However, this significant decline in viability was not observed when the complete treatment (including Triton) was performed. The addition of diamond particles to the chemical transformation did not negatively influence the viability, moreover the addition of either 5% DMSO or FNDs was shown to increase the viability. The electroporation protocol showed to be drastically affecting the cell viability (Fig. 3B) with a factor 100 lower compared to the control sample.

### Impact on cell morphology

**Figure 4:**
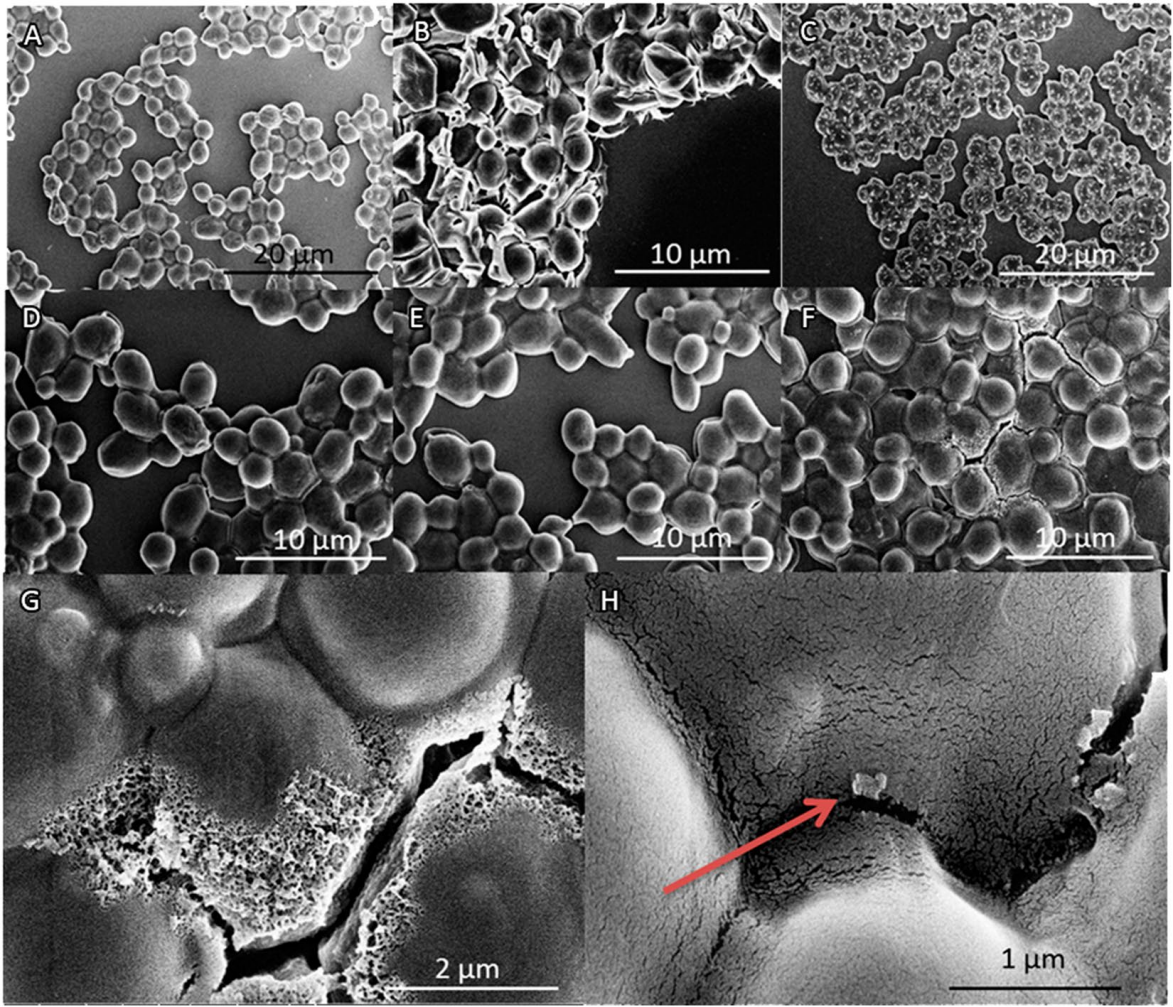
SEM visualizations of yeast cell topography and morphology. (**A**) Cells incubated with FNDs. (**B**) Cells after treatment with 2% DMSO and TMIX. Distorted cell morphology shows the side effects of this technique. (**C**) Cells treated with 0.1% Triton and FNDs: an accumulation of salts due to drying on the outside of the cells can be seen in white. (**D**) Cells treated with 0.1% Triton, TMIX and FNDs. (**E**) Cells electroporated with 1 pulse. Some light cellular damage can be observed. (**F**) Cells electroporated with 8 pulses. The cell wall of these cells is severely damaged as a result of the applied high electrical currents. (**G**) Disintegration of the cell wall results in puncturing and will lead to cell death (close up of F). (**H**) Close up of a diamond particle on the outside of a cell (indicated by the arrow, close up of A).

The morphology of Hxt6-GFP-expressing cells after transformation is shown in Fig. 4. Visual inspection revealed that the vast majority of cells remained intact after addition of FNDs (Fig. 4A), 2% DMSO and TMIX (Fig. 4B), FNDs and 0.1% Triton (Fig. 4C) and complete chemical transformation (4D). The crystals on the cells in Fig. 4B,C display remaining salt crystals from the drying process (salts contained in the medium). The transformation using electroporation resulted in damage to the cell wall (Fig. 4E–G). The most severely damaged cells can clearly be seen since they visibly lose cell wall integrity and cellular content (Fig. 4G, after 8 pulses electroporation). Nanodiamond particles on the surface could only rarely be seen in a few areas (Fig. 4H). The SEM results confirm the viability data qualitatively.

## Discussion

The forced internalization of nanodiamond sensors can prove extremely useful to study intracellular processes in yeast cells, or any other cells, which do not readily pick up diamonds. We have assessed a variety of protocols to overcome the thick cell wall obstacle. We show for the first time that yeast cells can take up fluorescent nanodiamond particles using a defined transformation mix combined with DMSO. Furthermore, we have evaluated the impact of different internalization protocols on cell viability. The techniques here are demonstrated in yeast cells, but will theoretically allow nanodiamonds to enter all kinds of cells with a cell wall, such as *E*. *coli* bacteria or plant cells.

The most effective and least-invasive method to induce fluorescent nanodiamond uptake in yeast cells is the use of a chemical transformation mix in combination with DMSO that weakens the cell wall. Electroporation can become an option if some of the reagents of the chemical transformation protocol are not compatible with the used cell. In this case the cellular response and its functioning should be closely monitored. Electroporation can also be useful when cell viability is secondary. This is for instance the case if one wants to detect how other cells react to (dead) yeast cells. Small numbers of cells with internalized FNDs or cells with a low number of internalized FNDs are useful for single cell studies. This is possible in the case of NV sensing, as this is usually done with one or very few particles. Having more internalized diamonds, however, is advantageous for labeling.

Although the method to quantify uptake does not count the exact number of diamond particles, we received a relative measure for the uptake. The method is designed in a way that membrane associated aggregates are excluded from the samples. This may have resulted in higher variation of the particles and in an underestimation of the number of internalized particles.

## Methods

### Cell cultures and materials

Experiments were performed using an Hxt6-GFP *S*. *cerevisiae* strain from the Yeast GFP Clone Collection from ThermoScientific^25^. This strain expresses a fusion of HeXose Transporter 6 (HXT6, a glucose transporter in the cellular membrane) with Green Fluorescent Protein, allowing the visualization of the cell membrane. Cells were grown overnight in synthetic dextrose complete medium (SD medium) at 30 °C, 200 rpm. Cellular concentration at the start of experiments was determined by measuring the optical density at 600 nm. 1.05 * 10^7^ cells/mL were used to return the cellular population to mid log phase (indicating the linear part of a logarithmic scale in the growth curve). Cells were then grown for an additional 2 h at 30 °C, 200 rpm. At the end of each experiment, cells were fixed in 3.7% paraformaldehyde for microscopic analysis. 1 M sorbitol was used as a buffer.

### Diamond particle characterization

The diamonds used in this study have an average diameter of 70 nm (FND70, Adamas Nano available as aqueous solution). They contain approximately 300 Nitrogen Vacancies per particle. They are produced by the manufacturer by grinding larger HPHT diamond and performing a selection of sizes. Since they are cleaned with oxidizing acids they have a carboxylated surface. Their fluorescence spectra are typical for NV centers with a broad emission of the phonon side band above 600 nm. Their recorded spectrum is shown in Supplementary Figure 1 and a schematic of the fluorescent mechanism of the diamond is shown in ref. 26 and in Supplementary Figure 2, reproduced from Nagl., *et al*.^27^.

### Trials to achieve uptake without permeabilizing the cell wall

Yeast cells were washed twice with H_2_O. Next, 2 µg of FNDs were added to the cells. FNDs were diluted with sorbitol to a total volume of 1 mL to avoid formation of FND aggregates. This mixture was then incubated for 2 hours at 30 °C to ensure maximum interaction between cells and diamonds. Under these conditions, no uptake can be expected so these samples served as negative controls. Next to this control condition, other physical and chemical options were tested to see if any influenced cellular uptake. First, instead of 1 M sorbitol as a solvent for the FNDs, H_2_O was used. Second, FNDs were coated with glucose before incubation. Since glucose can be utilized by yeast, they might have an incentive to ingest them. Third, FNDs were coated with FBS as described by Hemelaar *et al*.^5^ The glucose / FBS coated FND particles were made by incubating diamonds with a sterilized 0.1% w/v glucose solution or a 100% FBS-HI solution in a 1:10 ratio for several minutes. Fourth, cells were incubated in the dark instead of in the light. Finally, FNDs were incubated for 4 hours instead of 2. Internalized FNDs were found in none of the samples (see Supplementary Figure 3).

### Optimizing removal of diamonds on the cell surface

To prevent overestimating uptake, a detergent washing method to remove diamond particles from the cell surface was optimized. After adding 2 µg of FNDs to the washed yeast cells and incubating for 2 hours at 30 °C, three different detergents (Triton, Tween and SDS) were tested at concentrations of 1%, 0.5%, 0.1%, 0.05% and 0.01%. These detergents were added to the cell-FND suspensions and incubated for 30 min at RT. Removing surface diamonds reduces overestimation of uptake due to extracellular FNDs for microscopic analysis. Samples were compared to a non-washed suspension of yeast cells and diamonds. The most efficient detergent and concentration to wash diamond particles off the cellular exterior was Triton 0.01% (confirmed qualitatively by confocal microscopy, Supplementary Figure 4). In addition, the influence of the different detergents on the viability was tested. A concentration-dependent effect of the soap on the cell viability, tested using colony forming units, see below, was found for all soaps (Supplementary Figure 5). The 0.01% Triton solution was used in the chemical transformation protocol for further experiments.

### Chemical transformation TMIX

To achieve permeabilization of the yeast cells, we adapted and optimized a common protocol for chemical transformation of yeast cells from gene transfection. Mid log phase cells were washed in H_2_O and resuspended in 0.1 M LiAc in order to improve the chemical transformation efficiency^28^. The cells were spun down at 1000 × G and resuspended in H_2_O. The used transformation mix consists of 66.6% w/v PEG4000 and 1 M lithium acetate (LiAc). TMIX, FND_70_, DMSO 5% and 1 M sorbitol were added to yeast aliquots and the samples were incubated for 20 minutes at 42 °C. DMSO 5% was used to increase uptake efficiency and TMIX was added to weaken the cell wall. Afterwards the samples were washed with H_2_O and processed for microscopy or viability assays.

### Electroporation

Mid log phase cells were washed in H_2_O and resuspended in 0.1 M LiAc. The cells were spun down for 4 min at 1000xG and resuspended in sterile H_2_O. Aliquots of the yeast suspension were mixed with 2 µg FND_70_ in 1 M sorbitol solution. Electroporation was performed using an electroporation cuvette (Bio-Rad, Veenendaal, the Netherlands, catalog no. description 165/2089) and applying 1, 2, 4, 8, 12 or 16 pulses (25 µF/200 Ὠ/900 V) in a Bio-Rad Gene Pulser XcellTM Electroporation System, an adjustment of the electroporation protocol by E.L Rech *et al*.^29^ Afterwards the cells were centrifuged for 4 min at 1000xG and prepared for confocal microscopy by fixation or incubated on YPD plates (see below).

### Biocompatibility

To investigate the biocompatibility of the uptake and washing methods, colony forming units (CFUs) were counted after 48 hours of incubation at 30 °C on YPD plates (in triplicates) after a dilution series to prevent overcrowding. The respective controls for chemical and electrical transformation were produced with the exact same amount of pipetting and washing steps as for the transformation experiments. In order to check for cellular damage as a result of the used treatment, cells were imaged using SEM (FEI Magellan 400 XHR Scanning Electron Microscope, USA). In preparation, cells fixed in 1% glutaraldehyde and 4% paraformaldehyde in 0.1 M cacodylic acid were dried on a cover slip and coated with a 4 nm gold layer for 30 seconds at 20 °C, 40 mA in a Balzers SCD050 sputter coater.

### FND uptake quantification

Uptake was analyzed by fixing the cells in between a polylysine coated slide and a cover glass and imaging them using a Zeiss LSM 780 confocal laser scanning microscope (Zeiss, Germany). GFP was imaged at ex/em 488/525 nm and FNDs were imaged at 561/650 nm. To quantify the uptake of FNDs, the confocal images were analyzed by using the software Fiji (https://fiji.sc/). The Fiji protocol which has also been used in another study^8^ is described in the following. The analysis was divided into three phases: cell selection, masking and particle analysis. During the first phase, the images were visually inspected and 2 times approximately 100 random cells were selected for the analysis. Cells with large aggregates associated with the cell membrane were rejected post-hoc to prevent false positive results. The images were composed of several slices (z-stacks) and the cell’s region was defined in all the three dimensions. In the horizontal plane, the selection considered an area containing only the cell of interest. In the z-direction, the first and last slices containing the cell were manually identified using only the signal from the GFP channel to prevent biased selection. As a result, the first phase defines a volume that holds only the cell of interest. In the masking phase, that volume is molded in order to resemble the spheroid shape of the cell. The image is converted to binary (using the Isodata algorithm to calculate the threshold)^30^ and the cell’s perimeter is detected in every slice. To find the inner volume of the cell, the program shrinks the cell’s region in order to exclude the cell membrane from the analysis. The final step uses a special function of Fiji, which analyzes the particles found in a region. By applying this function to the masked image, it is possible to directly obtain the amount of objects (connected components) in the specified region. The performance of this process is set mainly by the parameter “threshold” which is used to separate the background light from the signal emitted by the FNDs. Every pixel with intensity less than the threshold is assumed as background and deleted from the image (set as black), while every pixel with an intensity greater than or equal to the threshold is assumed as part of a particle. To find an adequate value for this parameter, the image was visually inspected and different values were probed. Finally, the decision was made in favor of removing more background signal, but without deleting clearly identified particles. As a result, images containing no particles show no particles in the analysis. The process provides the number of objects, corresponding to the connected FND signal positive areas, and the number of particles, reflecting the actual amount of diamonds, calculated from the total intensity of the objects. After finding the right conditions for particle counting the same algorithm was applied to all conditions. Finally, in all samples the 10 cells with most particles located in the membrane regions were excluded, to prevent false positives from large aggregates at the surface.

### Embedding

Z-stacks in confocal microscopy give a good indication on the position of diamond particles. However, in order to be absolutely certain that diamond particles were inside the yeast cells, thin slices of the cell material were prepared. Finding diamond particles in slices taken in the middle of the cell confirms uptake. Samples fixed in 1% glutaraldehyde and 4% paraformaldehyde in 0.1 M cacodylic acid were resuspended in low melt agarose and cut to 1 mm^3^ sections. The samples were postfixed in 1% osmiumtetroxide/2.5% potassiumferrocyanide/0.1 M cacodylate buffer mixture for 2 hours. Next, the samples were dehydrated through an increasing graded ethanol series and left overnight in 1:1 ethanol and Epon (Serva) mixture at room temperature, which was replaced by pure Epon (4 times) and finally polymerized overnight at 58 °C^31^. After this, the samples were cut into semi-thin (approximately 0.8 µm) sections using an Ultracut E396879/M91103 microtome. Using a LSM780 confocal microscope, cell borders could be identified using differential interference contrast (DIC) and internalized nanodiamonds were detected using a 651 nm laser.

## Electronic supplementary material

Supplementary Information
