# Supplementary material for: Generally Applicable Transformation Protocols for Fluorescent Nanodiamond Internalization into Cells
Source: Sci Rep. 2017 Jul 19;7:5862. doi: 10.1038/s41598-017-06180-5 (PMC5517665; doi:10.1038/s41598-017-06180-5)
Supplement: Supplementary file 1 — Supplementary Information [file 41598_2017_6180_MOESM1_ESM.pdf]

Generally Applicable Transformation Protocols for Fluorescent Nanodiamond Internalization into Cells

Simon R. Hemelaar[1,+], Kiran J. van der Laan[1,+], Sophie R. Hinterding[1], Manon V. Koot[1], Else Ellermann[1], Felipe P. Perona-Martinez[1], David Roig[1], Severin Hommelet[1], Daniele Novarina[2], Hiroki Takahashi[3], Michael Chang[2], Romana Schirhagl[1]*

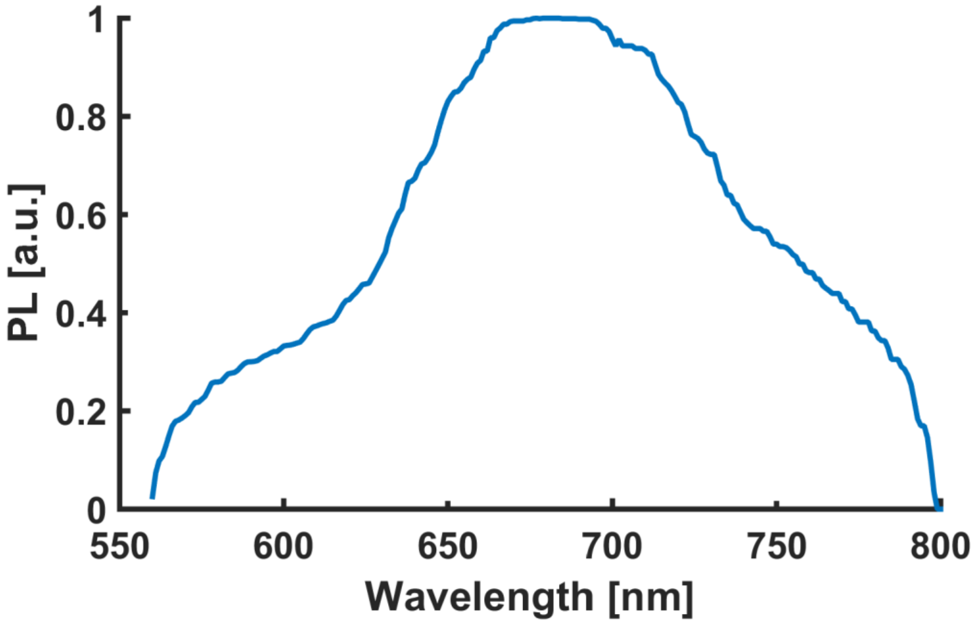

Supplementary Figure 1. Photoluminescent emission spectrum of 70nm FNDs measured at 532nm excitation (bandwidth 5nm) between 560 and 800nm. The spectrum was recorded using a Thermo Scientific Varioskan® Flash plate reader. Diamond emission was scanned with a wavelength step size of 1 nm and data was processed using the median filter function of MathLab software (http://www.mathlab.mtu.edu/mediawiki/index.php/Main_Page).

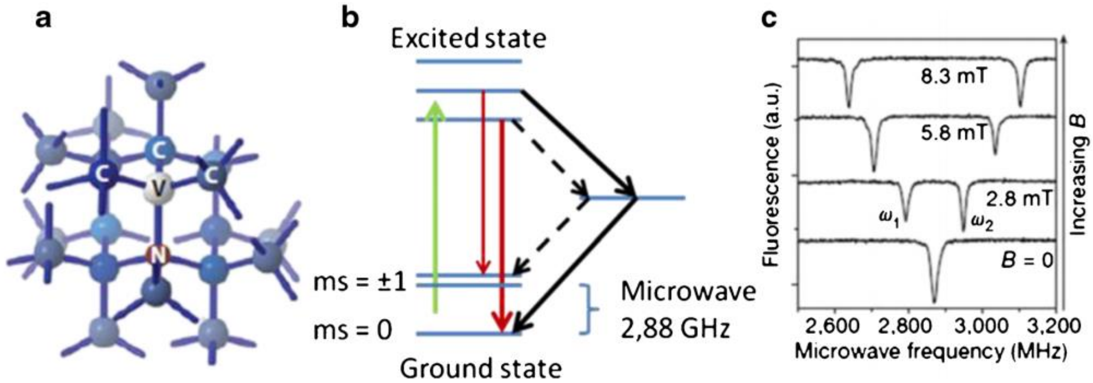

Supplementary figure 2. Optomagnetic properties of the NVcenter. **a** One carbon atom is replaced by a nitrogen next to a vacancy in the diamond lattice. **b** Simplified energy diagram of an NV-center. After excitation with a green laser, the NV-center emits red photons. If the electron is in the ms=±1 state, there is also an alternative way to the ground state over a dark state. As a result, fewer red photons are emitted and decreased fluorescence is observed. If a microwave is applied whose energy equals the difference between the

energies of the two states (2.88 GHz at zero field), the spins flip into the ms=±1 state. This effect can be observed as a drop in fluorescence (bottom curve in **c**). In presence of an external magnetic field the ms=±1 states are no longer equal in energy and thus split into two lines (top three curves in **c**). The difference is proportional to the field (Zeemann splitting), and the magnetic field can be determined. (**a** Reprinted with permission from [1]; **b** reprinted with permission from [2]; **c** reprinted with permission from [3]).

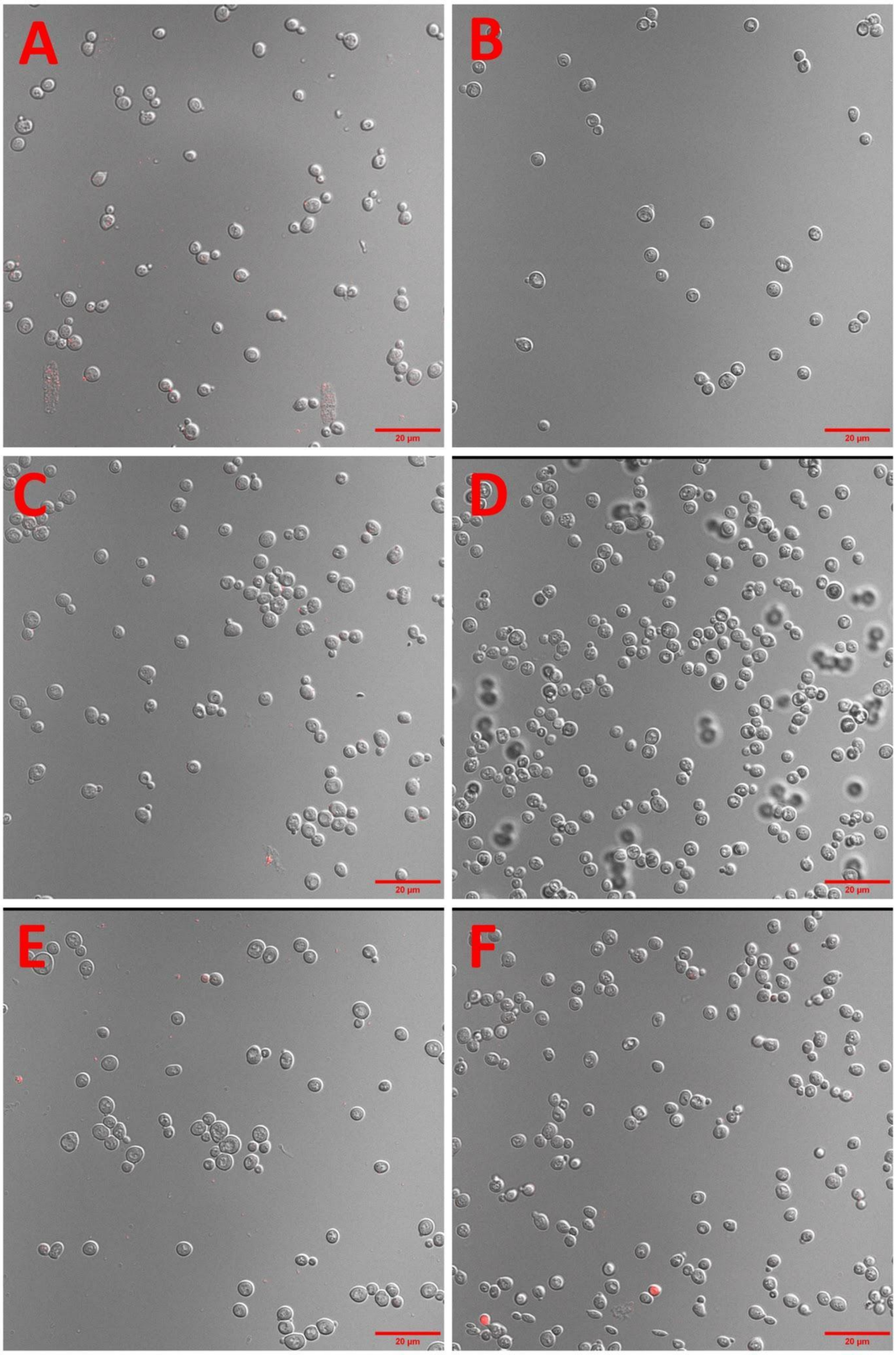

Supplementary Figure 3 Incubation conditions. Yeast cells were incubated with FNDs (2 µg/ml) in 1M sorbitol under different conditions for 2 hours at 30°C under different conditions without transformation, to show the non-uptake of yeast cells. A. The control sample. B. Instead of 1M sorbitol as a solvent for the FNDs, H_2O was used. C. FNDs coated with Glucose. D. Cells were incubated in the dark instead of in the light. E. FNDs coated with FBS. F. FNDs incubated for 4 hours instead of 2. In none of the samples internalized FNDs can be found. In some cases cells appeared positive for diamond, however we confirmed that this was background or the FNDs were stuck to the exterior of the cells.

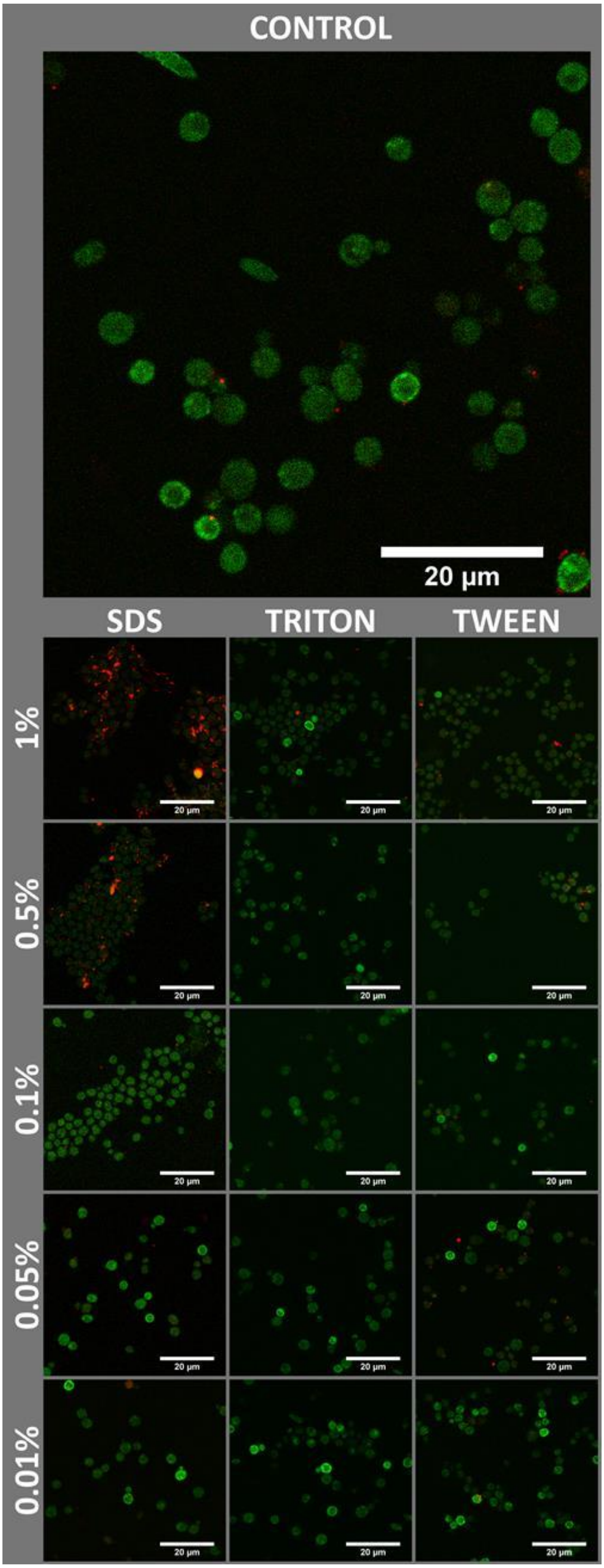

Supplementary Figure 4 Soap concentrations. We tested different soap concentrations of different soaps to see which would wash off externally bound FNDs the most efficiently. This appeared to be 0.01% Triton. Higher concentrations of SDS result in more extracellular bound FNDs. The SDS causes aggregation of the particles and at these high concentrations the cell wall is damaged, increasing the amount of binding sites for diamond (aggregates) of the particles which were still present in the sample.

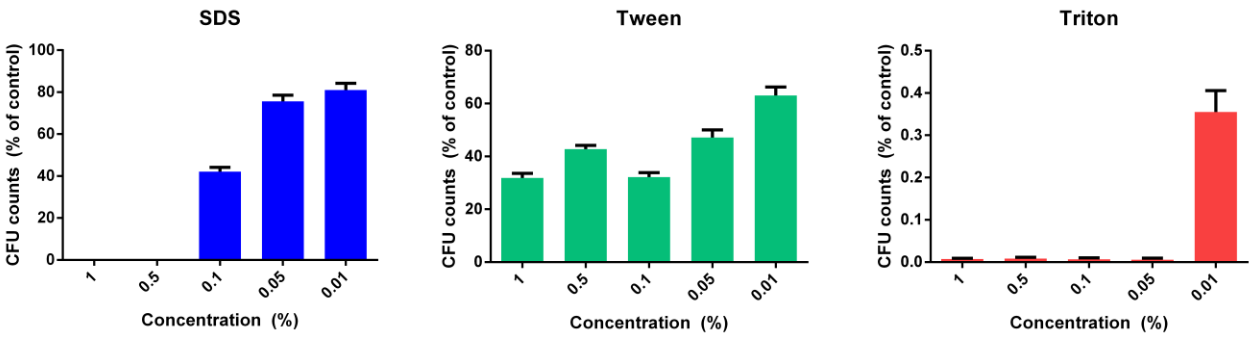

Supplementary Figure 5 Effect of detergents on viability. The different detergents used for removing particles from the cell surface, were tested for their effect on the viability of the cells. A concentration-dependent effect on the viability was found for all three soaps. SDS showed a strong negative influence on the cells at high concentrations. Tween had a minor effect on the cells even showing considerable amounts of viable cells at the highest soap concentration. Triton appeared to be the soap with the highest impact, decreasing the viability at all concentrations except the lowest (0.01%). To make sure that this low concentration was not an artifact, we also confirmed the viability to resemble normal values under even lower concentrations (0.001%, data not shown).

References

1. Schirhagl, R., Chang, K., Loretz, M. & Degen, C. L. Nitrogen-vacancy centers in diamond: nanoscale sensors for physics and biology. *Annu. Rev. Phys. Chem.* **65,** 83–105 (2014).

2. Nagl, A., Hemelaar, S. R. & Schirhagl, R. Improving Surface and Defect Center Chemistry of Fluorescent Nano-Diamonds for Imaging Purposes – A Review. *Anal. Bioanal. Chem.* (2015). doi:10.1007/s00216-015-8849-1

3. Balasubramanian, G. *et al.* Nanoscale imaging magnetometry with diamond spins under ambient conditions. *Nature* **455,** 648–651 (2008).
